# An 80-year-old man with a rare disease manifestation of disseminated cryptococcosis

**DOI:** 10.1016/j.idcr.2025.e02404

**Published:** 2025-10-15

**Authors:** Wouter L Smit, Laura M Vos, Gerdie M de Jong, Marloes J Schreuder, Lize F D van Vulpen, Ferry Hagen, Bart Vlaminckx, Marjolein P M Hensgens

**Affiliations:** a Department of Medical Microbiology, University Medical Center Utrecht, Utrecht, the Netherlands; b Department of Infectious Diseases, University Medical Centre Utrecht, Utrecht University, Utrecht, the Netherlands; c Department of Ophthalmology, University Medical Centre Utrecht, Utrecht University, Utrecht, the Netherlands; d Centre for Benign Haematology, Thrombosis and Haemostasis, Van Creveldkliniek, University Medical Centre Utrecht, Utrecht University, Utrecht, the Netherlands; e Westerdijk Fungal Biodiversity Institute, Utrecht, the Netherlands

**Keywords:** Cryptococcal infection, Non-HIV, Autoimmune hemolytic anemia, Panuveitis

## Abstract

We present an extraordinary manifestation of disseminated cryptococcosis in a man who developed a triad of acute panuveitis, autoimmune hemolytic anemia, and pulmonary cryptococcal infection. The patient exhibited progressive clinical deterioration with mediastinal lymphadenopathy, fever and severe hemolysis. Despite initial negative cryptococcal antigen testing, histopathological examination of a pulmonary nodule demonstrated yeast forms consistent with cryptococcal infection. The diagnosis was confirmed through molecular diagnostics and isolation of Cryptococcus neoformans from vitreous humor with subsequent positive antigen testing. No underlying immunocompromising disease was identified, although a low CD4+ count was found of unknown etiology. Therapeutic intervention comprised systemic high-dose fluconazole combined with intravitreal liposomal amphotericin B. This case exemplifies an unusual presentation of extrapulmonary cryptococcosis with isolated ocular involvement in the absence of meningitis, and suggests a potential association between cryptococcosis and autoimmune hemolytic anaemia.

An 80-year-old Dutch man was referred to our hospital because of acute panuveitis of his left eye and concurrent autoimmune hemolytic anemia (AIHA) of unknown origin. The patient’s medical history was notable only for glaucoma and no evidence of aging-associated conditions such as cardiovascular disease or prior malignancy. Over the past three months he suffered from fatigue and 4 kilos of unintended weight loss. He was a retired never-smoker and had no travel history outside Europe. In the secondary hospital, as part of diagnostic work-up for suspected cancer, a PET-scan was performed showing mediastinal FDG-avid lymphadenopathy up to 13 mm in size and a pulmonary nodule in the left upper lung (Fig. 1 A and B), from which a needle biopsy was taken. No malignancy was observed on histopathology. A Grocott's stain revealed multinucleated giant cells interspersed with round to oval thin-walled structures of 5–6 µm, suggestive of cryptococcosis (Fig. 1C). Two consecutive Cryptococcal serum antigen (CrAg) lateral flow assays were negative. A pan-fungal PCR test on aqueous humor was also negative, as well as blood and aqueous humor cultures. The patient received 1000 mg pulse-dose methylprednisolone and intravenous (IV) immunoglobulin for a presumed underlying autoimmune condition; however, due to a lack of therapeutic response he was transferred to an academic medical center for further diagnostic evaluation.

**Fig. 1:**
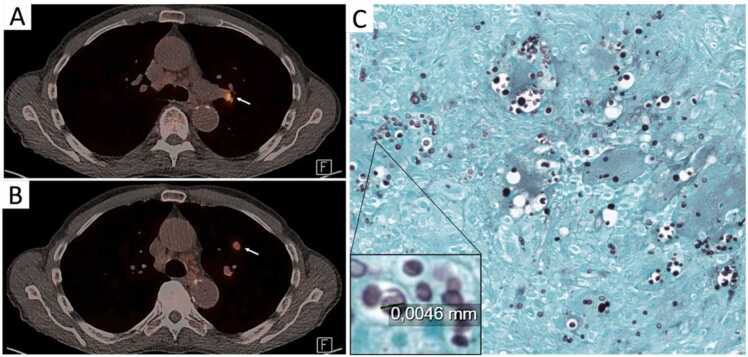
PET-scan of the patient showing (A) radiolucent hilar nodes and (B) a lesion in the left upper lung (right). (C) Grocott stain on pulmonary tissue.

Upon referral he presented with a hemoglobin level of 7.8 g/dL (ref: 13.8 – 17.2), positive Coombs test, presence of mixed warm and cold antibodies, and a high reticulocyte count of 277 × 10⁹/L (ref: 25 – 120 ×10⁹/L) He was lymphocytopenic (0.28 ×10⁹/L, ref: 0.8 – 4.0 ×10⁹/L) with a normal leukocyte count and mildly elevated C-reactive protein levels (12 mg/L, ref: –0,10). Erythropoietin, folic acid, and multiple blood transfusions were administered.

His left eye visual acuity was 0.10 decimal (20/200 Snellen) and intraocular pressure was 6 mmHg (ref: 10–21 mmHg). Slit-lamp examination revealed Descemet’s membrane folds with inferior keratic precipitates on the endothelium, anterior chamber cells with associated flare (no hypopion), and opacities with inflammation of the vitrious body. The optic disc was poorly visualized due to significant opacities and white exudates were observed in the inferior retina. B-scan showed intragel and retrogel floaters, multiple irregular retinal lesions and no signs of retinal detachment. This clinical picture, compatible with panuveïtis (Fig. 2A and B), was deteriorating after methylprednisolone pulses and discontinuation of antiviral therapy which was initiated at the referring hospital. This raised the suspicion of HSV/VZV-associated acute retinal necrosis and necessitated prompt initiation of systemic aciclovir and intravitreal foscarnet. Tuberculosis PCR on vitreous humor and interferon-gamma release assay were negative, and panuveïtis analysis showed no clues for a diagnosis. He developed episodes of fever and a decline in hemoglobin levels to 3.2 g/dL. A diagnostic pars plana vitrectomy was performed and a gram stain on vitreous humor showed presence of yeast cells (Fig. 2C).

**Fig. 2:**
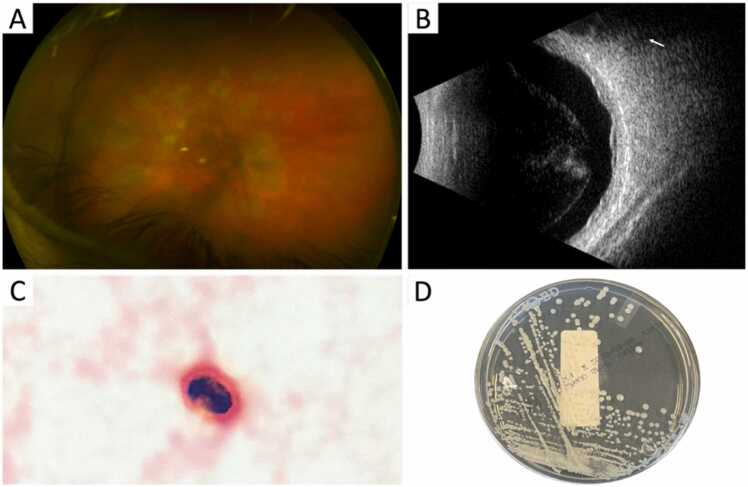
Microbiological evidence of disseminated pulmonary cryptococcosis to the eye. (A) Retinal exam showing multiple creamy yellow lesions in the posterior pole and midperiphery (B) Ultrasound B-scan showing intragel floaters fading at 88 dB, also retrogel floaters, multiple irregular retinal lesions, no signs of retinal detachment (C) Gram stain of vitreous humor showing growth of *C. neoformans* colonies on Sabouraud agar.

Our working diagnosis was disseminated cryptococcosis. We repeated the CrAg serum test which now showed a weakly reactive band interpreted as positive. An in-house pan-Cryptococcus species complex PCR on vitreous humor was positive, confirming the diagnosis of ocular cryptococcosis (cycle time of 31) [1]. After two days *C. neoformans* was isolated from vitreous humor, as determined by MALDI-TOF (Fig. 2D). Notably, there were no signs of meningeal involvement upon careful monitoring of neurological symptoms. A diagnostic lumbar puncture was carried out to rule out cryptococcal meningitis based on a negative CrAg test and PCR on cerebrospinal fluid. Treatment with high-dose oral fluconazole (1200 mg/day) was commenced and at day 7 of therapy local therapy with intravitreal liposomal amphotericin B injections (AmBisome®, 10 mcg/0.1 ml) was administered in addition to systemic therapy. Liposomal instead of conventional amphotericin B was used due to a favorable oculotoxicity profile. Systemic amphotericin B and flucytosine were considered but avoided due to concerns of bone marrow suppressive side effects in the context of AIHA. All antibiotics were discontinued and oral prednisone was tapered down to 30 mg daily. His blood levels gradually stabilized after 10 days of therapy and slit-lamp examination showed clear conjunctiva, no epithelial defect or corneal haze, and a clear anterior chamber (see table S1).

When searching for underlying risk factors for disseminated cryptococcosis, a CD4 + cell count of 39 cells/mm^3^ was detected. HIV was ruled out based on a negative HIV screening test at the referring hospital which was repeated at current hospitalization (Abbott Alinity i HIV Ag/Ab Combo assay of 4th generation). Prior to admission he did not use medication that could have impacted CD4 + cells, and a genetic cause seemed unlikely given his age and lack of a medical history of recurrent infections and normal immunoglobulin levels. Possibly methylprednisolone contributed by decreasing circulating CD4 + cells or, alternatively, it could be a late presentation of idiopathic CD4 lymphocytopenia. Although lymphadenopathy is described during disseminated cryptococcosis, enlarged lymph nodes could also indicate an underlying hematological malignancy or autoimmune disease (i.e. systemic lupus erythematosus) with associated lymphocytopenia. No evidence for these conditions was observed (lung biopsy did not show signs of malignancy, blood immune phenotyping did not show clonal proliferation, absence of a monoclonal M-protein). However, underlying diseases cannot be excluded, because after two weeks of medical treatment the patient refrained from both further therapy (including antifungal treatment) and additional diagnostic work-up (including investigation of lymph nodes or bone marrow investigation). The patient was transferred to receive hospice care and eventually deceased; no information about the cause of death could be provided.

Cryptococcosis is a basidiomycetous yeast that causes fungal infection of the lungs through inhalation of spores. Cryptococcus was originally classified into two species: *Cryptococcus neoformans* (varieties *grubii* and *neoformans*) and *Cryptococcus gattii*. However, recent phylogenetic analysis has identified seven distinct clades [2]. Disseminated cryptococcosis mostly affects immunocompromised patients, historically predominantly the HIV/AIDS population, causing life-threatening meningoencephalitis [3]. Bone (lytic lesions) and skin (molluscum-like lesions) involvement may occur in rare occasions. In the Netherlands, introduction of antiretroviral therapy led to a declining trend in the number of infections, whereas increasing use of immunomodulatory therapy has altered the epidemiological burden of cryptococcal meningoencephalitis which is now more concentrated in specific immunocompromised populations [4]. Other known risk factors are solid organ or hematopoietic stem cell transplantation, underlying autoimmune diseases, or in rare cases inborn errors of immunity [5]. Whereas *C. neoformans* is responsible for the large majority of infections in the immunocompromised host, *C. gattii* infections (endemic in (sub)tropical regions especially in Australia and parts of the Americas) have been documented more frequently in apparently healthy hosts [6]. However, recent evidence suggests that predisposing factors such as autoantibodies against granulocyte-macrophage colony-stimulating factor may underpin such cases [7], [8]. Presence of isolated pulmonary cryptococcoma in an otherwise asymptomatic patient may resolve without antifungal therapy [9], although withholding therapy is not recommended by current guidelines [10]. Ocular involvement usually constitutes choroiditis and papilledema which can be bilateral [11]. Although neuro-ophthalmologic findings in the context of cryptococcal meningitis in non-HIV patients have been well described, isolated ocular cryptococcosis without meningitis is highly unusual. In a small cohort of 44 previously healthy adults, headache was the main extra-ocular symptom (91 %), followed by brain abnormalities (such as leptomeningeal enhancement) on MRI (73 %), ventriculomegaly (43 %), and hydrocephalus (14 %) [11].

Mixed AIHA, characterized by erythrocyte destruction mediated by both IgM and IgG class autoantibodies, is precipitated by infections in approximately half of the cases while the remainder can be attributed to neoplastic or autoimmune etiologies [12]. AIHA associated with cryptococcosis has been reported: in such cases concomitant high-doses of prednisone used in AIHA therapy was considered to be the cause of cryptococcal disease [13]. We could not confirm whether AIHA was elicited by cryptococcosis, nor could we fully exclude the possibility of a non-infectious underlying condition causing lymphadenopathy, weight loss and AIHA. The patient's clinical deterioration and worsening anemia after IV methylprednisolone and immunoglobulin, which could have could have increased susceptibility to dissemination of cryptococcosis, combined with subsequent improvement observed after commencement of antifungal therapy suggest a potential infectious etiology for AIHA rather than an underlying auto-immune disease. However, a temporal relationship is obscured by the reintroduction of prednisone during the treatment course, complicating such conclusions. Also, the limited tissue from a needle biopsy may not entirely exclude a coexisting neoplastic process. Finally, it is worth mentioning that the initial CrAg serum test was negative whereas it was positive at a later timepoint. Potential causes are either a very low fungal load or, conversely, a postzone effect due to excess antigen [14].

Teaching points:

• Due to successful treatment of HIV, the burden of cryptococcal disease now concentrates in the non-HIV population in Western countries.
• Ocular involvement of extrapulmonary cryptococcosis is relatively rare, and patients usually present with concomitant signs of meningitis.
• Pulmonary cryptococcosis should prompt clinicians to investigate for signs of disseminated disease using the full array of diagnostics including serology, molecular testing, fungal culture, and pathology.
• This case suggests that disseminated cryptococcosis can be associated with severe autoimmune hemolytic anemia (AIHA).

## CRediT authorship contribution statement

**Laura M Vos:** Writing – review & editing. **Smit Wouter L:** Writing – original draft, Visualization, Resources, Conceptualization. **Marjolein PM Hensgens:** Writing – review & editing, Supervision. **Bart Vlaminckx:** Writing – review & editing. **Ferry Hagen:** Writing – review & editing, Resources. **Lize F D van Vulpen:** Writing – review & editing. **Marloes J Schreuder:** Writing – review & editing. **Gerdie M de Jong:** Writing – review & editing.

## Patient consent statement

Written informed consent was obtained from the patient for publication of this case report and any accompanying images. The case report was conducted in accordance with the ethical standards applicable in the country of origin and approved by the local institutional guidelines. No identifiable patient information is included in this report.

## Funding

The authors: no funding involved.

## Declaration of Competing Interest

The authors declare that they have no known competing financial interests or personal relationships that could have appeared to influence the work reported in this paper.
