# Supplementary figures and images for: An 80-year-old man with a rare disease manifestation of disseminated cryptococcosis
Source: IDCases. 2025 Oct 15;42:e02404. doi: 10.1016/j.idcr.2025.e02404 (PMC12557606; doi:10.1016/j.idcr.2025.e02404)

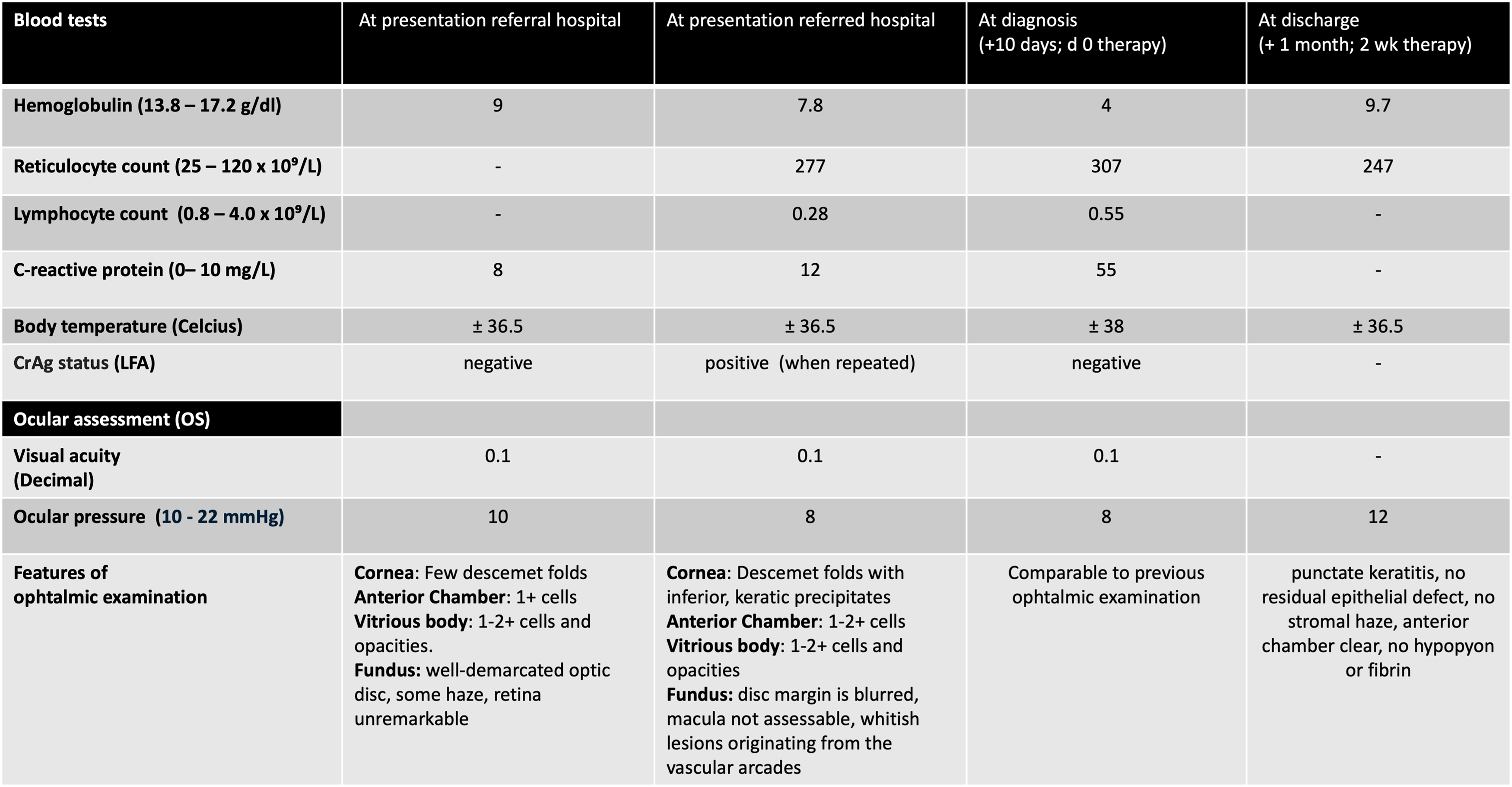

Supplement: Supplementary file 1 — Supplementary material [file mmc1.jpg]
